# Arg-73 of the RNA endonuclease MazF in *Salmonella enterica* subsp. *arizonae* contributes to guanine and uracil recognition in the cleavage sequence

**DOI:** 10.1016/j.jbc.2024.105636

**Published:** 2024-01-09

**Authors:** Takuma Okabe, Rie Aoi, Akiko Yokota, Hiroko Tamiya-Ishitsuka, Yunong Jiang, Akira Sasaki, Satoshi Tsuneda, Naohiro Noda

**Affiliations:** 1 Department of Life Science and Medical Bioscience, Waseda University, Tokyo, Japan; 2 Biomedical Research Institute, National Institute of Advanced Industrial Science and Technology (AIST), Ibaraki, Japan; 3 Graduate School of Comprehensive Human Sciences, University of Tsukuba, Ibaraki, Japan; 4 School of Integrative and Global Majors, University of Tsukuba, Ibaraki, Japan

**Keywords:** endoribonuclease, enzyme kinetics, fluorescence resonance energy transfer (FRET), protein engineering, RNA-protein interaction, *Salmonella enterica*, structural model, substrate specificity

## Abstract

The sequence-specific endoribonuclease MazF is widely conserved among prokaryotes. Approximately 20 different MazF cleavage sequences have been discovered, varying from three to seven nucleotides in length. Although MazFs from various prokaryotes were found, the cleavage sequences of most MazFs are unknown. Here, we characterized the conserved MazF of *Salmonella enterica* subsp. *arizonae* (MazF-SEA). Using massive parallel sequencing and fluorometric assays, we revealed that MazF-SEA preferentially cleaves the sequences U^∧^ACG and U^∧^ACU (^∧^ represents cleavage sites). In addition, we predicted the 3D structure of MazF-SEA using AlphaFold2 and aligned it with the crystal structure of RNA-bound *Bacillus subtilis* MazF to evaluate RNA interactions. We found Arg-73 of MazF-SEA interacts with RNAs containing G and U at the third position from the cleavage sites (U^∧^ACG and U^∧^ACU). We then obtained the mutated MazF-SEA R73L protein to evaluate the significance of Arg-73 interaction with RNAs containing G and U at this position. We also used fluorometric and kinetic assays and showed the enzymatic activity of MazF-SEA R73L for the sequence UACG and UACU was significantly decreased. These results suggest Arg-73 is essential for recognizing G and U at the third position from the cleavage sites. This is the first study to our knowledge to identify a single residue responsible for RNA recognition by MazF. Owing to its high specificity and ribosome-independence, MazF is useful for RNA cleavage *in vitro*. These results will likely contribute to increasing the diversity of MazF specificity and to furthering the application of MazF in RNA engineering.

The MazEF system is a type II toxin–antitoxin system widely conserved in bacteria and archaea as an addiction module (1, 2, 3). The MazEF system, originally discovered in *Escherichia coli* (3), consists of the antitoxin molecule MazE and the toxin molecule MazF. MazF in *E. coli* (MazF-ec) is normally suppressed by its cognate antitoxin molecule MazE-ec, and the two together form a complex called MazEF. During oxidative stress, UV irradiation, and antibiotics treatment, labile MazE-ec of the MazEF complex is degraded by ClpP and Lon proteases and MazF-ec gets activated (1, 4, 5, 6). Activated MazF-ec specifically cleaves ACA sequences in RNA, thus inhibiting translation (3) and resulting in a dormant state (6). To date, approximately 20 different MazF cleavage sequences, of three to seven nucleotides in length, have been found in bacteria and archaea. In different hosts, MazFs cleave different RNA sequences, and most MazFs have been reported to suppress bacterial activity. For example, overexpression of *Staphylococcus aureus* MazF (MazF-sa), which cleaves U^∧^ACAU (^∧^ represents cleavage sites) (7), inhibits cell division, and thickens cell walls (8). *Nitrosomonas europaea* MazF (MazF-ne1) preferentially cleaves the U^GG sequence, and as genes involved in energy production such as hydroxylamine oxidoreductase are UGG-rich (9), it is believed that MazF-ne1 inhibits cell growth by cleaving energy-producing genes under stress conditions (9). *Haloquadratum walsby* MazF, which cleaves the 7-nucleotide sequence UU^∧^ACUCA, may contribute to survival under hypoosmotic pressure by reducing pump expression (10).

MazF has physiological significance in stress responses, and it is an attractive tool for RNA engineering as it cleaves RNA in a sequence-specific manner. As MazF cleaves distinct RNA sequences in the absence of cofactors, including divalent metal ions (11), MazF-ec was used to determine mRNA integrity by LC-MS/MS. As it is difficult to quantify long mRNAs using LC-MS/MS, MazF-ec and two other endoribonucleases were used to generate cleaved mRNA (12). Utilizing multiple endoribonucleases with unique sequence specificities helps in improving the accuracy of mRNA sequence detection (12). The accuracy of mRNA sequences is essential for the development of oligonucleotide therapeutics, such as mRNA vaccines. As the demand for mRNA vaccines increases, insights into the diversity of MazF cleavage sequences will play a crucial role in the development of RNA therapeutics.

Nucleotide chain-bound *Bacillus subtilis* MazF (MazF-bs) and MazF-ec were crystallized to reveal their cleavage active sites and cleavage mechanisms (13, 14). Reports suggest that two sets of residues, Arg-25 and Thr-48 of MazF-bs and Arg-29 and Thr-52 of MazF-ec interact with RNA cleavage sites (13, 14). Further, the guanidinium group of Arg and the hydroxyl group of Thr act nucleophilically on the phosphate bond of RNA, causing transphosphorylation and forming a 2′3′-cyclic phosphate and 5′-hydroxyl group (14, 15). Arg is an essential residue for RNA cleavage activity; hence, when Arg-25 of *Candidatus* Desulforudis audaxviator MazF (MazF-da) was replaced with Ala, there was a complete loss in cleavage activity (16). Structural analysis also revealed an interaction between MazF residues and RNA bases recognized by MazF. MazF has a groove covered with three loops and α-helix, and these residues form hydrogen bonds and van der Waals bonds with RNA (13, 14). Hydrogen bonds are more common at the MazF cleavage sequence and less at the bases flanking the cleavage sequence (14). Notably, seven residues of MazF-bs (U^∧^ACAU) and MazF-ec (^∧^ACA) that form hydrogen bonds with the ACA sequence are conserved (14).

Mutations in conserved amino acids in MazF homologs with similar recognition sequences and RNA-recognizing amino acids alter MazF specificity. For example, MazF-da has been mutated at several conserved amino acids in MazF homologs with similar recognition sequences (16). Among these, mutation of Asp-36 in MazF-da resulted in an increase in the enzymatic activity for UACAAG compared to the WT (16). In addition, Park *et al.* used two chimeras to confirm their effects on MazF specificity for RNA (17). The loop of MazF-ec that interacts with RNA was replaced by the loop of two other MazFs that cleave a different sequence (17). Both chimeric MazFs were engineered to recognize the 5′ bases and recognized AAAAC, UUAAC, and UAAAC sequences in addition to the original ACA sequence (17). These studies suggest that the diversity of MazF specificity can be expanded by changing the amino acids that interact with RNA.

Recently, structure prediction models based on AlphaFold2, an open-source by DeepMind, have been used for structure analysis (18). In the 14th Critical Assessment of Protein Structure Prediction, AlphaFold2 recorded a median Global Distance Test Total Score of 92.4, which was remarkably higher than that of other competing teams (19). AlphaFold2 can accurately predict protein 3D structures with multiple sequence alignments and artificial intelligence within a few days (18). It can also be used *via* Google ColabFold to predict protein structures by submitting amino acid sequences (20). AlphaFold2 has been used to predict protein structures as an alternative to crystallization for structural analyses (21, 22, 23). Therefore, AlphaFold2 will help us better understand the cleavage function of MazF.

In this study, we evaluated the unknown cleavage sequence of MazF from *Salmonella enterica* subsp. *arizonae* (MazF-SEA), a human pathogenic bacterium. We then evaluated the cleavage mechanism of MazF-SEA using a 3D structure and identified the responsible residue for recognizing the base. In addition, MazFs were engineered by mutating the responsible residue of MazF-SEA or by introducing it into another MazF to confirm their effect on MazF specificity for RNA using the kinetic assay. This is the first study to identify a single responsible residue of MazF that is directly involved in RNA recognition using the AlphaFold2-predicted 3D structure. Our results can help engineer MazFs and contribute to increasing the diversity of MazF specificity.

## Results

### Enzymatic activity of MazF-SEA

**Figure 1:**
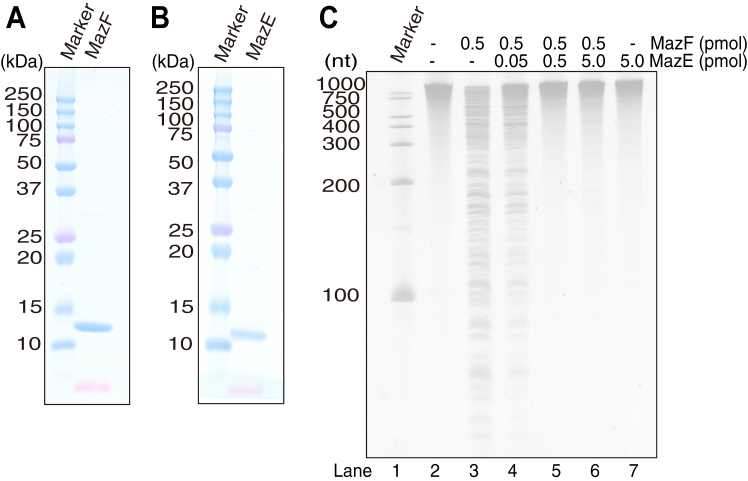
**Characterization of purified MazF-SEA and MazE-SEA**. Evaluation of molecular weights of (*A*) MazF-SEA and (*B*) MazE-SEA using SDS-PAGE. The theoretical molecular weights of MazF-SEA and MazE-SEA are 12.8 and 10.4 kDa, respectively. *C*, MazF-SEA cleavage ability was assessed using RNA and MazE-SEA. Lane 1, RNA ladder marker; Lane 2, substrate RNA (2000-1) with no enzyme; Lane 3, substrate RNA (2000-1) with 0.05 pmol of MazF-SEA; Lanes 4 to 6, substrate RNA (2000-1) with 0.05 pmol of MazF-SEA, which was preincubated with 0.05, 0.5, or 5.0 pmol of MazE-SEA; Lane7, substrate RNA (2000-1) with 5.0 pmol of MazE-SEA.

To investigate the enzymatic activity of MazF-SEA, MazF-SEA, and MazE-SEA were overexpressed in *E. coli* using isopropyl-β-D-thiogalactopyranoside (IPTG) and purified using affinity chromatography. Purified MazF-SEA and MazE-SEA were evaluated using SDS-PAGE and confirmed with their theoretical molecular weights, 12.8 kDa and 10.4 kDa, respectively (Fig. 1, *A* and *B*). Subsequently, artificial synthetic RNA (2000-1) with a length of 2033 nt was mixed with recombinant MazF-SEA. Artificial synthetic RNA that does not form a strong secondary structure has been previously employed for the MazF cleavage assay (24, 25). Synthetic RNA alone showed a single band (Fig. 1*C*, lane 2), whereas synthetic RNA with MazF-SEA exhibited fragmented bands (Fig. 1*C*, lane 3), confirming the RNA cleavage activity of recombinant MazF-SEA. Further, MazF-SEA and MazE-SEA were mixed and reacted with synthetic RNA. On adding MazE-SEA (0.05 pmol) to MazF-SEA (0.5 pmol), the RNA fragmentation decreased slightly (Fig. 1*C*, lane 4). When MazE-SEA was added in equal or greater amounts than MazF-SEA, the RNA cleavage activity of MazF-SEA was completely suppressed (Fig. 1*C*, lanes 5 and 6). This confirmed that MazE-SEA inhibited the RNA cleavage activity of MazF-SEA in a dose-dependent manner. These results indicate that MazF-SEA is a ribonuclease and MazE-SEA is a cognate antitoxin of MazF-SEA.

### Cleavage sequence identification

**Figure 2:**
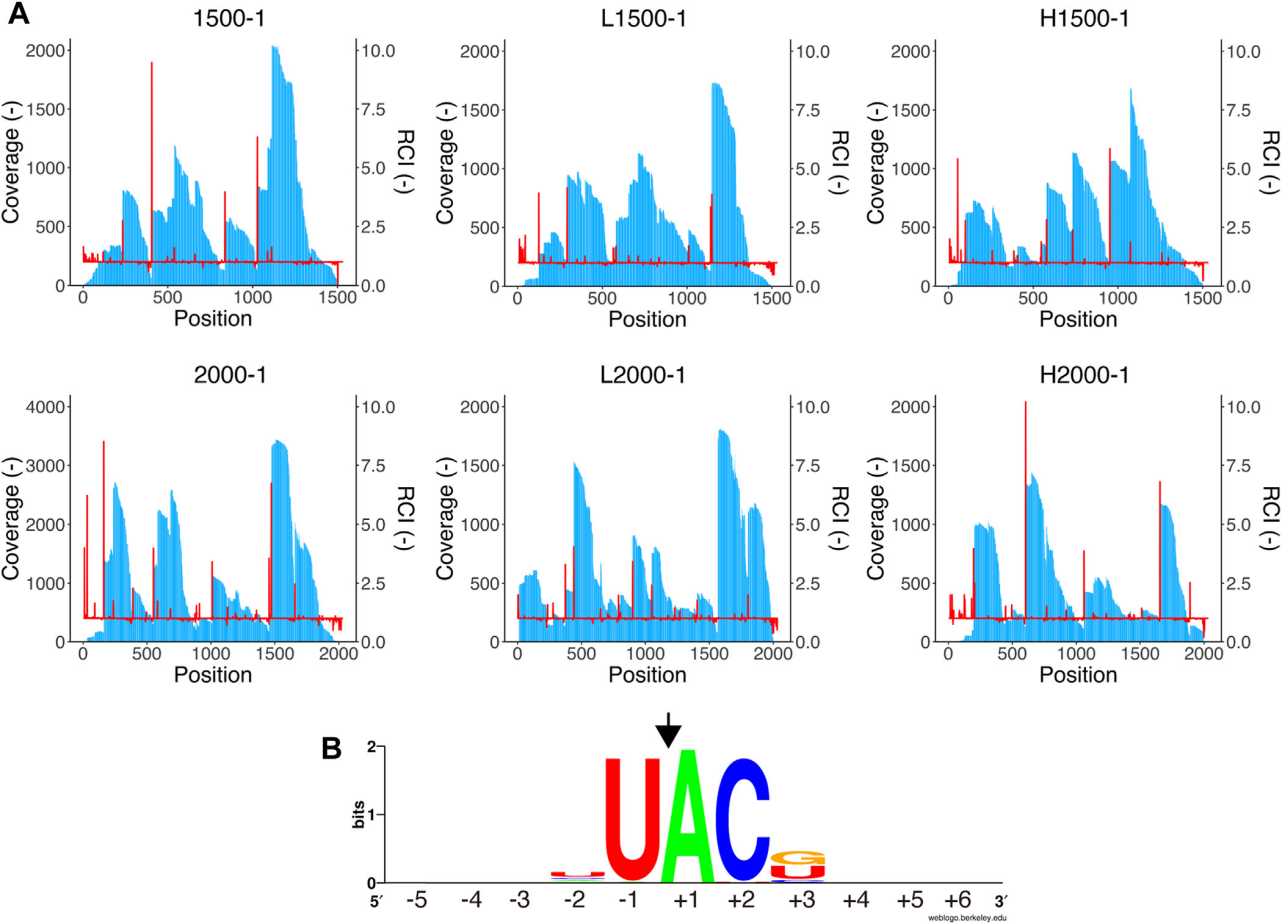
**Identification of MazF-SEA cleaving sequence**. *A*, the values of coverage (blue bar) and relative coverage increase (RCI; red line) are shown at the base of each graph. *B*, the base frequencies of the cleavage sequences were visualized using WebLogo. Bases at position +1 are one base beyond the cleavage site and are defined as RCI > 1.5 and Coverage > 50. The *black arrow* represents the cleavage site. The sequence number refers to the number of bases away from the cleavage site as 0.

The cleavage sequence of MazF-SEA was characterized using massive parallel sequencing (25). Six synthetic RNAs (1500-1, L1500-1, H1500-1, 2000-1, L2000-1, and H2000-1) consisting of artificial sequences were cleaved using MazF-SEA, followed by ligating barcoded RNA to the 5′-end of the cleaved RNA. cDNA was then synthesized from the barcode-ligated RNA and sequenced using the MiSeq. Reads harboring the barcode RNA sequences were mapped to the sequences of six synthetic RNAs. This allowed for a surge in coverage and relative coverage increase (RCI) values at the bases after the cleavage sites (Fig. 2*A*). Fifty-eight bases with coverage of more than 50 and RCI of more than 1.5 were extracted as bases following the cleavage sites (Table S1). These bases including 5 nt upstream and 5 nt downstream were aligned, and base frequencies were verified using WebLogo (26). Consequently, U^∧^ACG and U^∧^ACU emerged as candidate MazF-SEA cleavage sequences (Fig. 2*B*). Since MazF-SEA was found to predominantly recognize and cleave the serial four nucleotides (U^∧^ACG and U^∧^ACU), 58 sequences were further investigated to elucidate the number of U^∧^ACN. Of the 58 sequences, 49 contained U^∧^ACG or U^∧^ACU, and 7 contained U^∧^ACC or U^∧^ACA (Table S1). Thus, MazF-SEA mainly cleaved U^∧^ACG and U^∧^ACU, but also slightly cleaved U^∧^ACC and U^∧^ACA.

**Figure 3:**
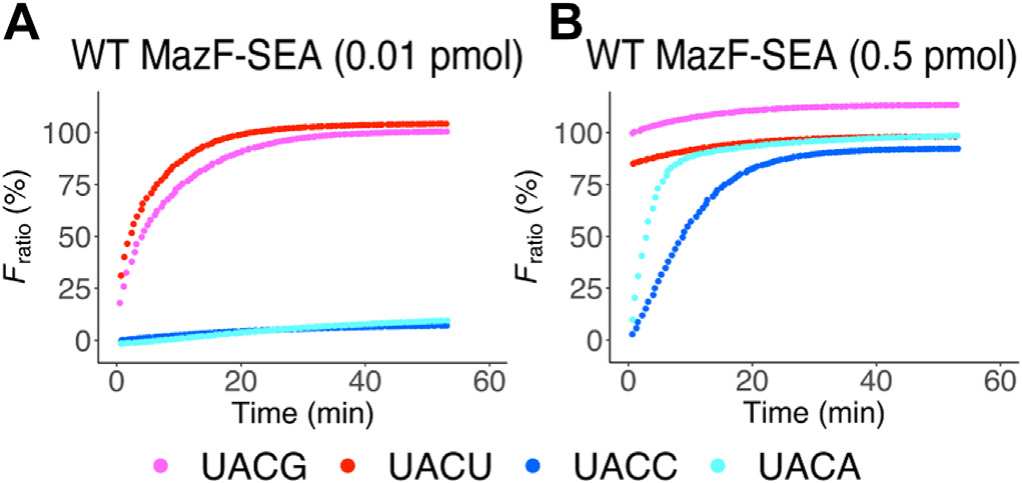
**Fluorometric assay of WT MazF-SEA.** RNA/DNA chimeric oligonucleotides containing UACG (*pink*), UACU (*red*), UACC (*blue*), or UACA (*cyan*) were mixed with (*A*) 0.01 pmol of WT or (*B*) 0.5 pmol of WT.

To determine the enzymatic activity of MazF-SEA for U^∧^ACG, U^∧^ACU, U^∧^ACC, and U^∧^ACA, a fluorometric assay was performed. The fluorometric assay uses a short RNA/DNA chimeric oligonucleotide probe modified with 6-carboxyfluorescein (6-FAM) at the 5′ end and black hole quencher-1 (BHQ-1) at the 3' end as a substrate, which emits fluorescence when the probe is cleaved by MazF (27). In this assay, RNA/DNA chimeric oligonucleotide probes containing UACG, UACU, UACC, or UACA were incubated with MazF-SEA at 37 ºC, followed by fluorescence intensity measurement. The fluorescence intensities increased logarithmically when UACG or UACU probes were mixed with 0.01 pmol of MazF-SEA (Fig. 3*A*). In contrast, the fluorescence intensities did not increase when UACC or UACA probes were mixed with 0.01 pmol of MazF-SEA (Fig. 3*A*); however, when 0.5 pmol of MazF-SEA was added, the fluorescence intensities increased logarithmically (Fig. 3*B*). Further, when 0.5 pmol of MazF-SEA was mixed with UACG or UACU probes, the fluorescence intensities reached the maximal value, even at t = 0 (Fig. 3*B*). In addition, when MazF-SEA was mixed with MazE-SEA, MazE-SEA inhibited cleavage activities of WT MazF-SEA in a dose-dependent manner (Fig. S1, *A*–*D*). Thus, the cleavage of the probes was due to recombinant MazF-SEA. In conclusion, MazF-SEA showed high enzymatic activity for UACG and UACU and low enzymatic activity for UACC and UACA.

### Prediction of interactions between MazF-SEA and RNA

**Figure 4:**
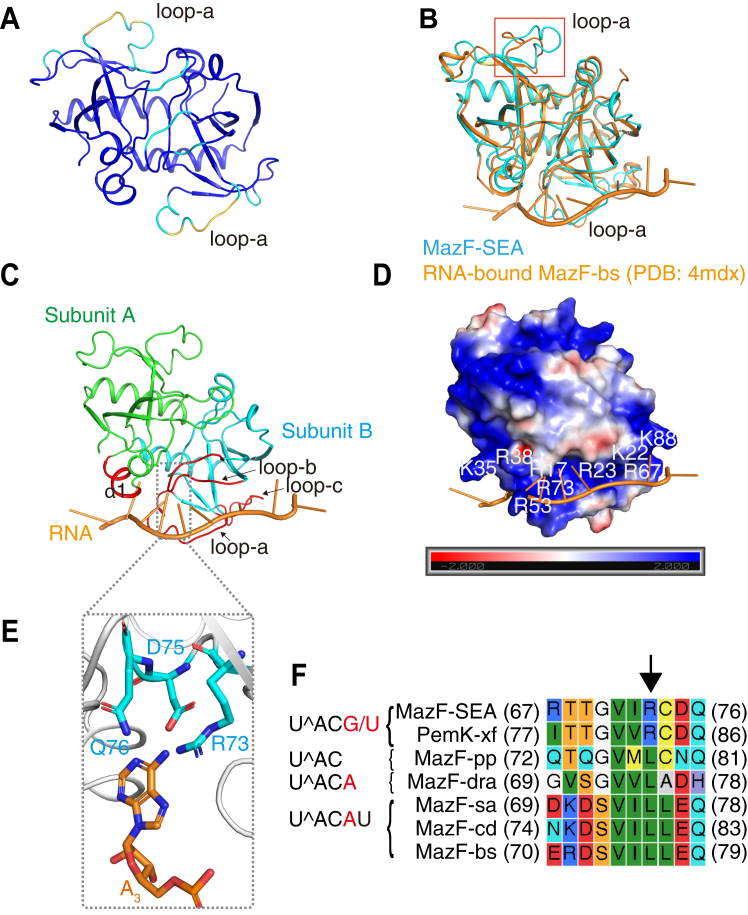
**Prediction of the interaction between MazF-SEA and RNA**. *A*, ColabFold predicted the 3D structure of MazF-SEA as a homodimer. The color represents pLDDT score (*blue*: >90, *cyan*: 70–90, *yellow*: 50–70, and *red*: <50), no red-colored residues were observed. Some residues (^48^GGN^50^ and A^52^) in loop-a of MazF-SEA were colored *yellow*. *B*, MazF-SEA (*cyan*) was aligned with a co-crystal structure of RNA (U_-3_U_-2_dU_-1_A_1_C_2_A_3_U_4_A_5_A_6_)-bound MazF-bs (orange, PDB:4mdx). The loop-a in opposition to the RNA binding site, which was highlighted with a *red box*, has a significant structural difference between MazF-SEA and MazF-bs. *C*, RNA-bound MazF-SEA model. The RNA structure (*orange*) is extracted from the co-crystal structure of RNA-bound MazF-bs (4mdx) and superposed on the MazF-SEA model. The color represents each subunit of MazF-SEA (*green*: subunit A and *cyan*: subunit B). In addition, the predicted RNA binding site, which is composed of one α-helix (α1) in subunit A and three loops (loop-a, loop-b, and loop-c) in subunit B of MazF-SEA, was colored in red. *D*, RNA-bound MazF-SEA model, whose electrostatic surface was visualized. The charge on the MazF-SEA surface was colored according to electrostatics. The scale bar showed the electrostatic values from −2.0 kT/e (*red*) to 2.0 kT/e (*blue*). RNA (*orange*) was placed in a positively charged groove. Nine residues contributing to positive charge were shown in the figure. *E*, zoom up where MazF-SEA is assumed to interact with the third base from the cleavage site (A_3_). A_3_ indicates the third base from the cleavage site of MazF-bs. The side chains of three residues, which were mainly adjacent to A_3_, are shown. *Red and blue* represent the oxygen atoms and the nitrogen atoms, respectively. *F*, primary sequences of MazF homologs that cleave similar sequences as MazF-SEA were aligned with that of MazF-SEA using CLC Genomics Workbench 12.0.1. *Xylella fastidiosa* PemK (PemK-xf) cleaves U^∧^ACG and U^∧^ACU (28), *Pseudomonas putida* MazF (MazF-pp) cleaves U^∧^AC (25). *Deinococcus radiodurans* MazF (MazF-dra) cleaves U^∧^ACA (29). MazF-sa, MazF-bs, and *Clostridium difficile* MazF (MazF-cd) cleaves U^∧^ACAU (7, 30, 31). The sequence identity between MazF-SEA and MazF homologs was calculated to be 46% for PemK-xf, 47% for MazF-pp, 33% for MazF-dra, 28% for MazF-sa, 27% for MazF-bs, and 26% for MazF-cd. The left-most sequence in the figure is the cleavage sequence of each MazF and red alphabets represent the third base from the cleavage site. The *black arrow* represents the position of Arg-73 of MazF-SEA. Each number in parentheses represents the residue number of MazF.

The residues of MazF-SEA that recognize G and U at the third base from the cleavage sites were determined using the 3D structure and multiple sequence alignment. Since MazF acts as a homodimer, we predicted the 3D structure of MazF-SEA as a homodimer using ColabFold (20), which can access AlphaFold2 (18). While AlphaFold2 predicts the protein 3D structure, it outputs the predicted Local Distance Difference Test (pLDDT) on a scale of 0 to 100 for each residue as a confidence score (18). A score above 90 indicated high confidence, 70 to 90 indicated intermediate confidence, 50 to 70 indicated low confidence, and below 50 indicated no confidence (18). The pLDDT scores of the 92 residues in MazF-SEA (110 amino acids) were above 90, which are colored blue in Figure 4*A*, and none of the residues had a pLDDT score below 50. However, the pLDDT scores for some residues in loop-a (^48^GGNFARTAG^56^) were between 50 and 70 (Fig. 4*A*). MazF-bs, already crystallized as an RNA-bound complex, cleaves an RNA sequence (U^∧^ACAU) like MazF-SEA (U^∧^ACG/U). Crystallization analysis of MazF-bs revealed that the loop (^50^QIQKAK^55^) of MazF-bs, equivalent to loop-a of MazF-SEA, changed its conformation when MazF-bs bonded with RNA (13). Thus, some residues in the loop-a of MazF-SEA may change their conformation upon binding to RNA and result in lower pLDDT scores.

The 3D structural model of MazF-SEA was aligned with the cocrystal structure of RNA (U_-3_U_-2_dU_-1_A_1_C_2_A_3_U_4_A_5_A_6_)-bound MazF-bs (PDB: 4mdx) (Fig. 4*B*). Despite only 27% sequence homology between MazF-SEA and MazF-bs, their structural conformations were similar, except for a loop-a on the opposite side of the RNA binding site (Fig. 4*B*, red box). The RNA-bound MazF-SEA model was constructed (Fig. 4*C*) by concealing only the MazF-bs structure from Figure 4*B*. One α-helix (α1) in subunit A and three loops (loop-a, loop-b, and loop-c) in subunit B of MazF-SEA are assumed to interact with RNA (Fig. 4*C*). These structures had a positively charged groove on the RNA interacting site (Fig. 4*D*) and the groove had nine positively charged residues (His-17, Lys-22, Arg-23, Lys-35, Arg-38, Arg-53, Arg-67, Arg-73, and Lys-88) (Fig. 4*D*). As RNA is negatively charged, the positively charged MazF-SEA groove easily bonded with the RNA. We evaluated the interaction between MazF-SEA and its cleavage sequence (Fig. S2). The U_-1_^A_1_C_2_ was a common cleavage sequence found in both MazF-SEA (U_-1_^A_1_C_2_G_3_/U_3_) and MazF-bs (U_-1_^A_1_C_2_A_3_U_4_), and the residues interacting with U_-1_^A_1_C_2_ were highly conserved. Since the Ser-73 side chain of MazF-bs mainly interacts with dU_-1_ (13), Thr-68, or Thr-69, which are similar to Ser, MazF-SEA is assumed to interact with dU_-1_ (Fig. S2). Residues of MazF involved in cleavage are generally Arg and Thr (13, 14, 16), and MazF-SEA also possessed similar Arg-23 and Thr-46 residues (Fig. S2). In MazF-bs, the main chains of Gly-18, Glu-20, and Gly-22 interacted with A_1_, and in MazF-SEA, the main chains of Gly-16 and Glu-18 and the side chain of Ser-20 are assumed to interact with A_1_ (Fig. S2). In MazF-bs, Ser-19 and Gln-21 interacted with C_2_, while in MazF-SEA, His-17 and Gln-19 are assumed to interact with C_2_ (Fig. S2). Contrastingly, the cleavage sequences of MazF-SEA (U_-1_^A_1_C_2_G_3_/U_3_) and MazF-bs (U_-1_^A_1_C_2_A_3_U_4_) differed in the third base from the cleavage site, where MazF-SEA recognized G_3_ or U_3_, while MazF-bs recognized A_3_. Focusing on the third base from the cleavage site (A_3_) in the RNA-bound MazF-SEA model, the side chains of Arg-73, Asp-75, and Gln-76 were adjacent to A_3_ (Figs. 4*E* and S2). The pLDDT scores of the three residues were above 90; hence, these side-chain orientations had high confidence. The Arg-73 side chain of MazF-SEA was particularly positioned at the coordinates closest to A_3_ (Fig. 4*E*). Under the experimental conditions of RNA cleavage at pH 8.0, Arg is positively charged and contains a guanidinium group, whereas G and U, recognized by MazF-SEA at the third base from the cleavage site, commonly have ketone groups. Thus, the guanidinium group of Arg-73 might form hydrogen bonds with the ketone groups of G and U. Furthermore, the amino acid sequences of MazF-SEA and other MazF homologs that cleave sequences like MazF-SEA were aligned (Fig. 4*F*). *Xylella fastidiosa* PemK (PemK-xf) cleaves U^∧^ACG/U (28). *Pseudomonas putida* MazF (MazF-pp) cleaves U^∧^AC (25). *Deinococcus radiodurans* MazF (MazF-dra) cleaves U^∧^ACA (29). MazF-sa, MazF-bs, and *Clostridium difficile* MazF (MazF-cd) cleave U^∧^ACAU (7, 30, 31). Arg-73 was conserved in MazF-SEA and PemK-xf cleaving U^∧^ACG and U^∧^ACU, whereas Leu was conserved in MazF homologs cleaving U^∧^AC, U^∧^ACA, or U^∧^ACAU (Fig. 4*F*). Hence, we concluded that Arg-73 of MazF-SEA plays a key role in recognizing G and U at the third base from the cleavage site.

### Arg-73 of MazF-SEA contributes specificity for the third base from cleavage sites

**Figure 5:**
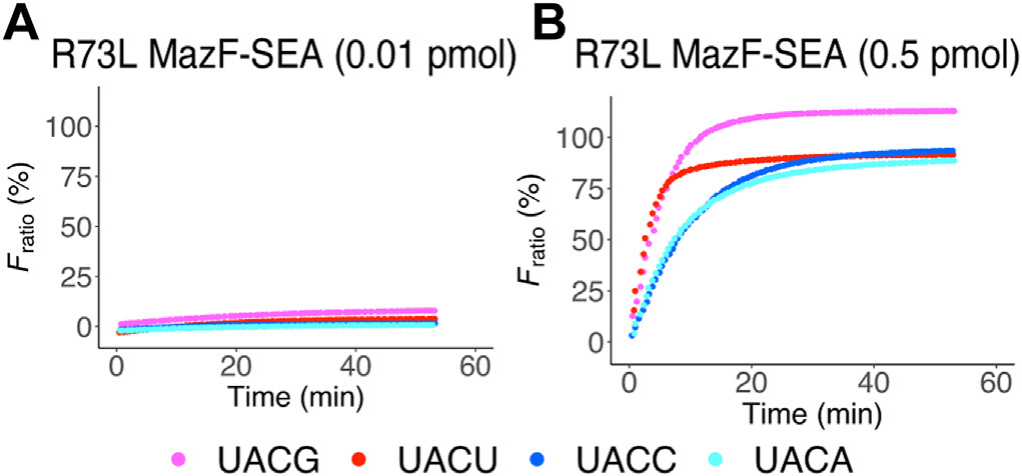
**Fluorometric assay of R73L mutant MazF-SEA**. RNA/DNA chimeric oligonucleotides containing UACG (*pink*), UACU (*red*), UACC (*blue*), or UACA (*cyan*) were mixed with (*A*) 0.01 pmol of R73L mutant or (*B*) 0.5 pmol of R73L mutant.

MazF-SEA R73L mutant was expressed and purified (Fig. S3*A*). We conducted a fluorometric assay to evaluate the specificity of the R73L mutant for UACG and UACU. The resulting fluorescence intensities did not increase when 0.01 pmol of R73L mutant was mixed with either UACG, UACU, UACC, or UACA probes (Fig. 5*A*). However, the fluorescence intensities increased logarithmically when 0.5 pmol of R73L mutant was mixed with the probes (Fig. 5*B*). Compared to the WT, the cleavage activity of R73L mutant decreased against UACG and UACU, but not against UACC and UACA.

**Figure 6:**
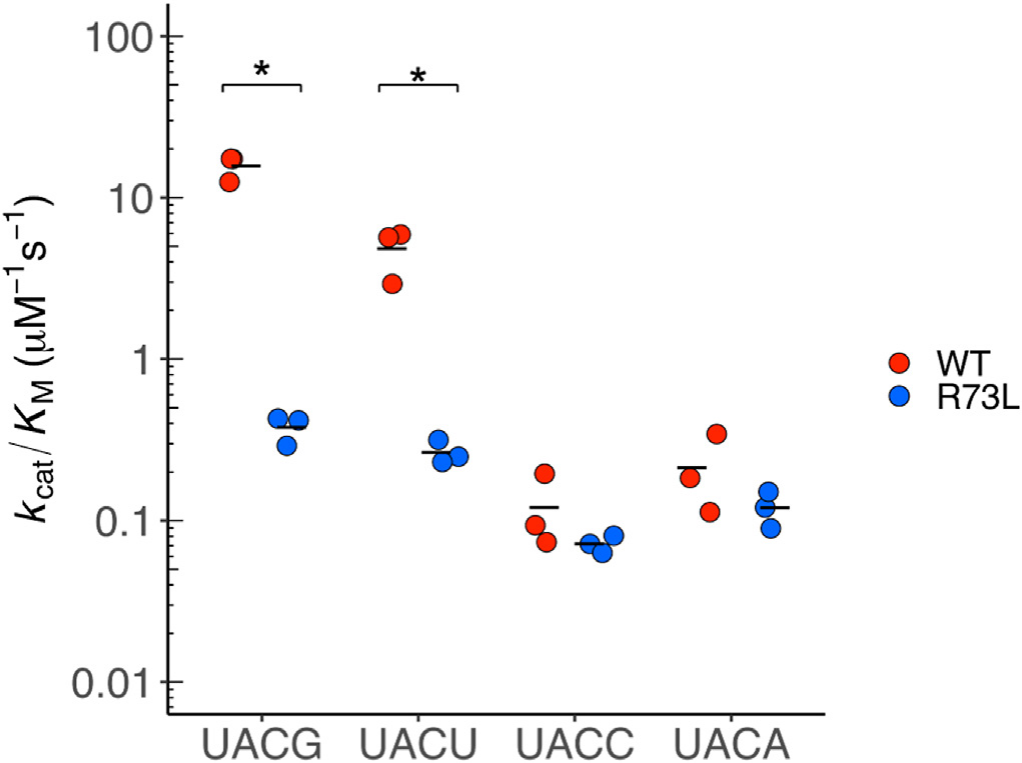
**Catalytic efficiency (k**_**cat**_**/K**_**M**_**) of WT and R73L mutant MazF-SEA for UACG, UACU, UACC, or UACA**. *Red and blue plots* represent WT and R73L mutants, respectively. *Asterisks* indicate *p*-values smaller than 0.01 (*p* < 0.01). The *black bars* represent an average of three catalytic efficiencies for each substrate.

**Figure 7:**
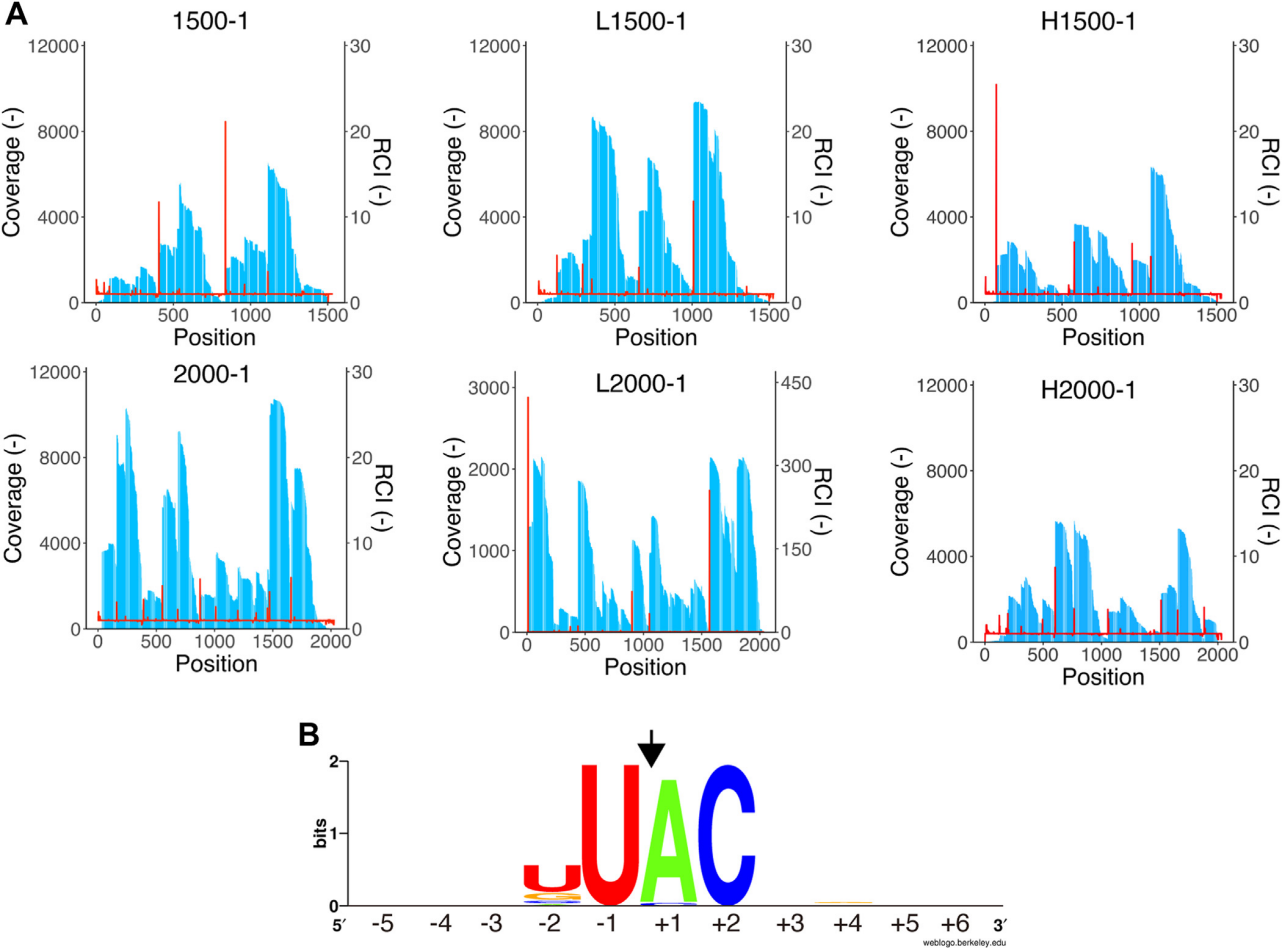
**Identification of R73L mutant MazF-SEA cleavage sequences**. *A*, values of coverage (*blue bar*) and relative coverage increase (RCI; *red line*) are shown at the base of each graph. *B*, base frequencies of the cleavage sequences were visualized using WebLogo. Bases at position +1 are one base beyond the cleavage site and are defined as RCI > 1.5 and Coverage > 50. The *black arrow* represents the cleavage site. The sequence number refers to the number of bases away from the cleavage site as 0.

The enzyme kinetics of WT and R73L mutant MazF-SEA were evaluated using the UACG, UACU, UACC, and UACA probes. Catalytic efficiencies (*k*_cat_*/K*_M_) of WT for UACG and UACU were 16 and 4.8 μM^−1^s^−1^, respectively (Fig. 6 and Table S2). In contrast, *k*_cat_*/K*_M_ of the R73L mutant for UACG and UACU reduced considerably to 0.38 and 0.27 μM^−1^s^−1^, respectively (Fig. 6 and Table S2). Compared to the WT, the *k*_cat_*/K*_M_ of the R73L mutant was lower by 42-fold and 18-fold for UACG and UACU, respectively. Conversely, the *k*_cat_*/K*_M_ of the R73L mutant for UACC and UACA was equivalent to WT (Fig. 6). Thus, the R73L mutant had a remarkably lower catalytic efficiency for UACG and UACU compared to that of WT, but not in the case of UACC and UACA. Furthermore, focusing on the R73L mutant against UACG, UACU, UACC, and UACA, there were no significant differences in their respective catalytic efficiencies (0.072–0.38 μM^-1^s^-1^). This result indicated that the specific base was no longer recognized at the third position from the cleavage site. Subsequently, the cleavage specificity of R73L mutant was determined using massive parallel sequencing with six synthetic RNAs (1500-1, L1500-1, H1500-1, 2000-1, L2000-1, and H2000-1). Sixty-one bases that exhibited coverage of more than 50 and RCI of more than 1.5 were extracted as bases following the cleavage sites (Fig. 7*A* and Table S3). When 61 sequences were aligned using WebLogo, U^∧^ACN emerged as a candidate for the R73L mutant cleavage sequences (Fig. 7*B*). Of 61 sequences, 19 contained U^∧^ACG, 14 contained U^∧^ACU, 13 contained U^∧^ACC, and 13 contained U^∧^ACA (Table S3). In conclusion, the Arg-73 to Leu mutation in MazF-SEA reduced the enzymatic activity for UACG and UACU only.

Further, MazF-SEA R73E and R73K mutants were engineered to alter specificity. R73E mutant slightly cleaved the UACG only at 0.5 pmol (Fig. S4*A*), but 0.5 pmol of R73K mutant did not cleave any probes (Fig. S4*B*), even though Lys resembles Arg in properties. Thus, replacing Arg-73 with Glu or Lys reduced the cleavage activity.

### Introduction of Arg-73 into another MazF

**Figure 8:**
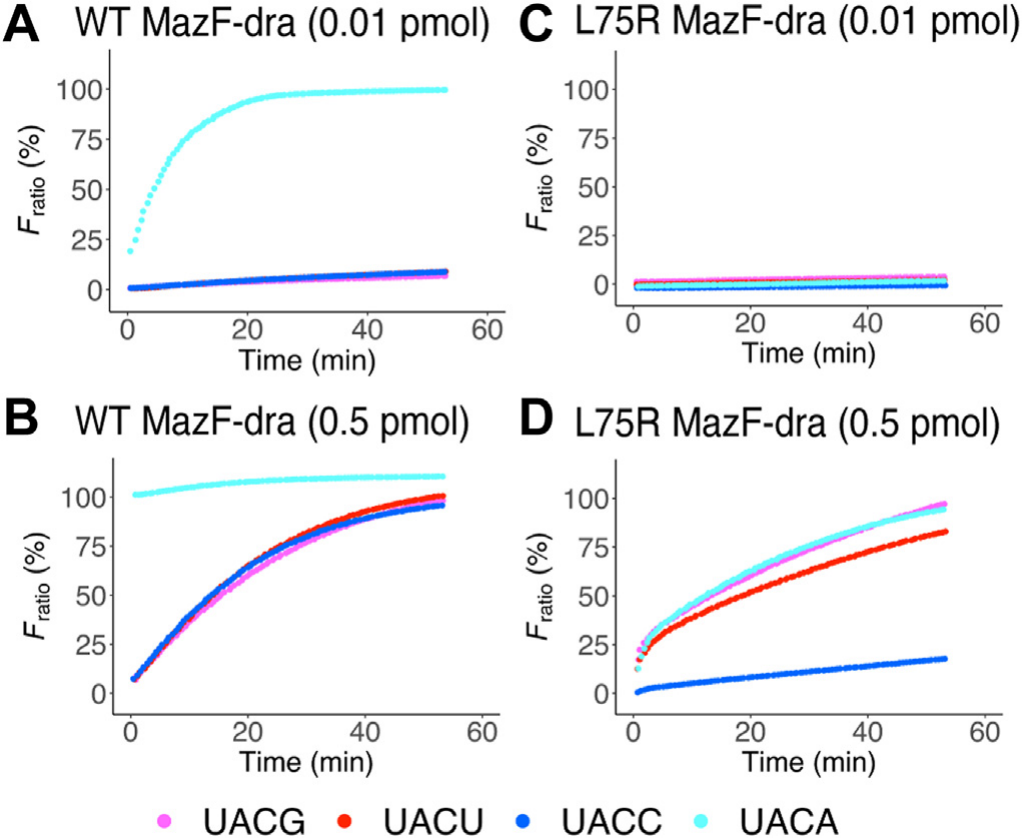
**Fluorometric assay of WT and L75R mutant MazF-dra.** RNA/DNA chimeric oligonucleotides containing UACG (*pink*), UACU (*red*), UACC (*blue*), or UACA (*cyan*) were incubated with (*A*) 0.01 pmol of WT, (*B*) 0.5 pmol of WT, (*C*) 0.01 pmol of L75R mutant, or (*D*) 0.5 pmol of L75R mutant.

Arg-73 in MazF-SEA is an essential residue that recognizes G and U at the third base from the cleavage site. To evaluate the performance of Arg-73 in other MazFs, Arg-73 was introduced into other RNA-cleaving MazFs. We employed MazF-dra, which specifically cleaves U^∧^ACA but not U^∧^ACG and U^∧^ACU (29). Leu-75 of MazF-dra was equivalent to that of MazF-SEA Arg-73 (Fig. 4*F*). WT and the L75R mutant MazF-dra were expressed in *E. coli* and purified (Fig. S3, *B* and *C*). The WT MazF-dra was incubated with the UACG, UACU, UACC, or UACA probes and only the fluorescence intensities of the UACA probe increased logarithmically at 0.01 pmol concentration of WT MazF-dra (Fig. 8*A*). This result was consistent with the results of a previous study (29). When 0.5 pmol of WT MazF-dra was added, it cleaved UACG, UACU, and UACC (Fig. 8*B*). Although the cleavage activity of WT MazF-dra on UACG was not confirmed in a previous study (29), the cleavage activity of the WT on UACG was like that of the WT on the UACU and UACC (Fig. 8*B*). L75R mutant MazF-dra was also incubated with UACG, UACU, UACC, or UACA probes. The fluorescence intensities did not increase when 0.01 pmol of L75R mutant MazF-dra was incubated with any of the probes (Fig. 8*C*). These results showed that L75R mutant MazF-dra reduced the enzymatic activity for UACA (Fig. 8, *A* and *C*). In contrast, the fluorescence intensity increased logarithmically when 0.5 pmol of L75R mutant MazF-dra was mixed with UACG, UACU, or UACA, but not with UACC (Fig. 8*D*). Compared to WT, the mutation reduced the cleavage activity against UACA to the same extent as against UACG and UACU, and cleavage activity against UACC to the extent that 0.5 pmol of MazF could not cleave it. The mutation reduced the cleavage activity against UACA and UACC, whereas it remained unchanged against UACG and UACU. The unchanged cleavage activity of UACG and UACU was assumed to be due to the Arg residue mutation; thus, Arg substitution contributed to the recognition of G and U at the third base from the cleavage site.

## Discussion

*S. enterica* subsp. *arizonae*, classified as a non-typhoidal *Salmonella*, resides in the intestines of reptiles (32, 33). Most infections involving reptiles, as pets or as food, can be avoided, but immunocompromised people, due to underlying conditions, get infected and their condition deteriorates (32, 33). As MazF has been proposed to regulate virulence genes in pathogenic bacteria (7), it is important to consider the intracellular function of MazF-SEA in *Salmonella*. According to the kinetic assay results, the main cleavage sequences of WT MazF-SEA were UACG and UACU (Fig. 3*A*). The U_-1_^A_1_C_2_ was a corresponding cleavage sequence found in both MazF-SEA (U_-1_^A_1_C_2_G_3_/U_3_) and MazF-bs (U_-1_^A_1_C_2_A_3_U_4_). Although the sequence identity between MazF-SEA and MazF-bs was only 27%, five common residues (Gly-16, Glu-18, Gln-19, Arg-23, and Thr-46 of MazF-SEA) interacted with the common cleavage sequence U^∧^AC (Fig. S2). A structural comparison of nucleic acid-bound MazF-ec and nucleic acid-bound MazF-bs showed that seven common residues formed hydrogen bonds with the corresponding cleavage sequence ^ACA (14), while the sequence identity between MazF-ec and MazF-bs was only 24% (14). In addition, the RNA interactions between MazF-dra and MazF-bs were compared in a previous study (34). Although MazF-dra and MazF-bs had only 21% sequence identity, eight common interactions with the corresponding cleavage sequence (U^∧^ACA) were identified (34). Thus, the cleavage sequences are expected to be identical if the residues around the RNA binding site are conserved, even with low sequence identity among MazF homologs. In MazF-bs, Lys and Gln residues in the movable loop (^50^QIQKAK^55^), which is equivalent to loop-a (^48^GGNFARTAG^56^) of MazF-SEA, changed conformation inward or outward during RNA binding (13). Positively charged or hydrophilic amino acids may change their conformations during RNA binding. Hence, Asn-50, Arg-53, and Thr-54 in the loop-a of MazF-SEA would contribute to conformational changes when MazF-SEA binds to RNA. This conformational change also affected the prediction in ColabFold, resulting in lower pLDDT scores for several residues in loop-a of MazF-SEA. In a previous study, when Arg-54 of MazF-dra was mutated to Ala, the enzymatic activity of MazF-dra decreased (34), thus Arg-53 in MazF-SEA was assumed to play a role in RNA attachment. This confirms that the Arg residue in the movable loop of MazF is important for RNA capture.

The substitution of Arg-73 with Leu in MazF-SEA resulted in comparable catalytic efficiencies for UACG, UACU, UACC, and UACA. R73L mutant showed properties like those of MazF-pp. MazF-SEA is phylogenetically similar to MazF-pp (47.4% identity) and PemK-xf (45.9% identity). However, MazF-SEA cleaves U^∧^ACG/U and MazF-pp cleaves U^∧^ACN. If MazF-pp and MazF-SEA were derived from the same ancestor, MazF may have gained or lost Arg-73 as *P. putida* and *S. enterica* adapted to their respective environments. Although our results are of considerable evolutionary interest, it is unclear how the MazF RNA recognition sequences change over time, and it requires further study. In the present study, we attempted to modify the specificity of MazF-SEA by mutating Arg-73 to Glu, which significantly reduced the enzyme activity. Luscombe *et al.* investigated the universal preference of DNA–protein interactions; for example, Arg and Lys prefer G, and Glu prefers C, suggesting that charged amino acids are more likely to form hydrogen bonds (35). However, we found that R73E mutant MazF-SEA could not be modified to recognize C at the third base from the cleavage sequence. In addition, although Arg and Lys have similar properties, the R73K mutant significantly reduced cleavage activity. This could be attributed to the lengths of the Arg and Lys side chains. Because Arg is the longest-charged amino acid, replacing it with another residue hindered the interaction with RNA. Therefore, the enzymatic activities of R73E and R73K mutants MazF-SEA were significantly reduced. Arg residues are responsible for a variety of enzymatic activities, including γ-glutamyl group transfer (36) and realization of supercoiled DNA (37); Arg is also generally required for MazF homologs, including MazF-SEA, for RNA digestion (13, 14, 16). The 73rd Arg residue of MazF-SEA plays an essential role in the recognition of G and U residues in the RNA strand. Enzymes that cleave nucleic acids other than MazF, such as restriction enzymes and RNases, have also been reported to have specific residues responsible for base recognition. Arg-119 of the restriction enzyme BtsI recognizes the first base G of the GCAGTG sequence; when Arg-119 is mutated to Ala, the specificity of the first base is lost (38). In RNase Mini-III, mutation of N93R changes its specificity from ACCU (specificity of the WT) to AUCU (39). Thus, Arg is a vital residue of microbial enzymes that are involved in base recognition and catalysis.

In addition to MazF, various RNA-cleaving enzymes have been reported to be toxin molecules in the toxin-antitoxin systems. RelE, YoeB, HigB, and YafO, which are toxin molecules of type II toxin-antitoxin systems, cleave ribosome-binding RNA in a sequence-specific manner (40, 41, 42, 43). Compared to these RNA-cleaving enzymes, MazF can easily cleave RNA *in vitro* in a ribosome-independent manner, allowing for sequence-specific cleavage of RNA by simply mixing the substrate RNA and MazF. A ribosome-independent endoribonuclease HicA has no sequence specificity for mRNA (44), whereas MazF is highly specific for RNA sequences, recognizing 3 to 7 nucleotides in length. AbiQ and ToxN, the toxin molecules of type III toxin-antitoxin systems, specifically cleave adenine-rich RNA sequences (45, 46). However, they have only been studied in a few bacterial species, hence it is uncertain whether they have the same sequence-specific diversity as MazF. Although there are several RNA-cleaving enzymes in the toxin-antitoxin systems, MazF was utilized to evaluate mRNA integrity by LC-MS/MS because it can cleave single-stranded RNA in a ribosome-independent manner and is readily available *in vitro*. Furthermore, because it is difficult to examine long mRNAs using LC-MS/MS, the use of multiple MazFs with unique sequence specificities helps in improving the accuracy of mRNA sequence detection (12). Approximately 20 cleavage sequences of MazF have been discovered, and MazF is highly diverse among RNA-cleaving enzymes. This study shows that the introduction of Arg residues into MazF has the potential to expand the diversity of RNA substrate specificity. Thus, we believe that this study will lead to improvements in mRNA mass spectrometry analysis technology which requires sequence diversity for RNA cleavage.

MazF-SEA from *S. enterica* cleaves U^∧^ACG and U^∧^ACU in the RNA sequence and Arg-73 directly recognizes G and U at the third base from the cleavage site. This is the first study to identify a single characteristic residue of MazF that is directly involved in RNA recognition using the AlphaFold2-predicted 3D structure. This finding contributes to the understanding of MazF substrate specificity and the MazF evolution study. The discovery and development of MazF's diverse cleavage sequences, like those of restriction enzymes, will be widely used in RNA measurement and other areas of RNA engineering. However, there is a lack of information on the interactions of MazF with RNA required for its specificity modification. To this end, it is necessary to further investigate the RNA recognition mode of several MazFs.

## Experimental procedures

### Plasmids, RNAs, and oligonucleotides

pET21a(+) vectors encoding *mazF* or *mazE* were obtained from GenScript. The gene sequences were optimized for overexpression in *E. coli.* Three 1533 nt (1500-1, L1500-1, and H1500-1) and three 2033 nt (2000-1, L2000-1, and H2000-1) synthetic RNAs were prepared. The 45 nt-barcode RNA (Table S4) was constructed by Japan Bio Services. All RNA/DNA chimeric oligonucleotides that harbor 6-FAM at the 5′-end and BHQ-1 at the 3′-end were constructed by Japan Bio Services (Table S4).

### Protein expression and purification

*E. coli* BL21(DE3)pLysS (BioDynamics Laboratory) was transformed with pET21a-*mazF*-SEA (WT, R73L, or R73E mutants). *E. coli* BL21(DE3) (Nippon Gene) was transformed with pET21a-*mazE*-SEA, pET21a-*mazF*-SEA-R73K, or pET21a-*mazF*-dra (WT or L75R mutant). These cells were grown at 37 ºC in 1 L of LB medium until OD_600_ exceeded 5.0, followed by 1 mM of IPTG to induce MazF or MazE expression. After 3 h of induction, the cells were collected by centrifugation at 7500*g* for 5 min and stored at −80 °C until purification. Defrosted cells were resuspended with degassed binding buffer (pH 8.0, 20 mM sodium phosphate buffer, 300 mM NaCl, 40 mM imidazole, 5 mM β-mercaptoethanol, and 0.05% Triton X-100), and were sonicated for 20 min with Handy Sonic UR-20P (Tomy Digital Biology). Supernatants were collected by centrifugation at 5100*g* for 10 min. These solutions were filtered with 0.45 μM membrane (Merck) and applied to 1 ml His Trap FF crude column (Cytiva). The column was washed with 45 column volumes of the binding buffer using AKTA Pure 25 (Cytiva). Thereafter, MazF-SEA and MazE-SEA were eluted by steadily adding elution buffer (pH 8.0, 20 mM sodium phosphate buffer, 300 mM NaCl, 500 mM imidazole, 5 mM β-mercaptoethanol, and 0.05% Triton X-100). A part of purified proteins was denatured at 95 ºC for 3 min and confirmed by SDS-PAGE. The protein concentration was measured using a Bio-Rad Protein Assay (Bio-Rad).

### Enzymatic activity of MazF-SEA and neutralization by MazE-SEA

As the substrate, 0.45 pmol of the synthetic RNA (2000–1) was incubated with 0.05 pmol of MazF-SEA at 37 ºC for 10 min in 30 μl of MazF reaction buffer (40 mM Tris-HCl (pH 8.0), 1 mM dithiothreitol, 0.01% Triton X-100, and 4 U Recombinant RNase inhibitor (Takara)). In addition, 0.05, 0.5, and 5.0 pmol of MazE-SEA were incubated with 0.05 pmol of MazF-SEA at 25 ºC for 10 min and incubated with 0.45 pmol of the synthetic RNA (2000–1). Incubated RNA was purified using RNA Clean and Concentrator-5 (Zymo Research) and then incubated at 95 ºC for 5 min with gel-loading dye II (Ambion). The denatured RNA was separated on a 10% polyacrylamide gel containing 7 M urea. The RNA was stained with SYBR Gold (Life Technologies) and detected using a Typhoon 9210 imager (Cytiva).

### Cleavage sequence identification

The MazF-SEA cleavage sequence was identified as described previously (25). Six synthetic RNAs (1500-1, L1500-1, H1500-1, 2000-1, L2000-1, and H2000-1) were cleaved with 0.25 pmol of MazF-SEA or 1.0 pmol of R73L mutant MazF-SEA at 37 °C for 10 min in MazF reaction buffer and purified with RNA Clean and Concentrator-5 (Zymo Research). Phosphorylation, barcode RNA ligation, and sequence library construction were performed as previously described (25). Sequencing was performed on a MiSeq platform using the MiSeq reagent kit v2 (500-cycles, Illumina). Sequencing data were analyzed using CLC Genomics Workbench 12.0.1 (Qiagen). Barcode-ligated RNAs were extracted and mapped to six synthetic RNAs using parameters, as described previously (25). The RCI was calculated as the coverage of the n^th^ nucleotide divided by the coverage of the (n-1)th (n ≥ 2). If the coverage of the (n-1)th is 0, a pseudo count of 1 was inserted and RCI was calculated. Subsequently, bases that exhibited RCI of more than 1.5 and coverage of more than 50 were chosen as MazF cleavage sites, and sequences from 5 nt upstream to 5 nt downstream were aligned with WebLogo (26). The sequence data of massive parallel sequencing were submitted to the DDBJ database under accession numbers DRA015035 and DRA015036.

### Fluorometric assay

In 20 μl of MazF reaction buffer, 0.01 and 0.5 pmol of MazF-SEA (WT or R73L mutant) or MazF-dra (WT or L75R) were reacted with 20 pmol of the RNA/DNA chimeric oligonucleotide probes containing either UACG, UACU, UACC, or UACA at 37 °C. Fluorescence intensity was recorded every 30 s using a Light Cycler 480. The excitation and detection wavelengths were 465 nm and 510 nm, respectively. In addition, 100 ng of RNase A (Novagen) and no enzyme were mixed with 20 pmol of each probe as positive and negative controls, respectively. Subsequently, the fluorescence intensity of the MazF-cleaved oligonucleotide (*F*_MazF_) was normalized to that of the positive control (*F*_positive_) and negative control (*F*_negative_) using the following equation (Equation 1).

(1) $$\begin{array}{c} F_{ratio}=\frac{\left(F_{MazF}-F_{negative}\right)}{\left(F_{positive}-F_{negative}\right)}\times 100 \end{array}$$

### Prediction of protein 3D structure

The structure of MazF-SEA was predicted with ColabFold (DeepMind). This tool (https://colab.research.google.com/github/sokrypton/ColabFold/blob/main/AlphaFold2.ipynb) uses MMseq2 for multiple sequence alignments to predict the protein structure through AlphaFold2 (DeepMind). Five models were predicted using ColabFold, which also calculated pLDDT and predicted the aligned error (pAE). The five models were ranked in order of pAE. The highest rank of the model was used for analysis. The predicted MazF-SEA was aligned to the co-crystal structure of RNA-bound MazF-bs using the PyMOL Molecular Graphics System 2.4.1 (Schrödinger).

### Determination of kinetic parameters

The kinetic parameters (*k*_cat_/*K*_M_) of MazF-SEA (WT and R73L mutant) were determined using RNA/DNA chimeric oligonucleotide probes containing UACG, UACU, UACC, or UACA. First, the suitable concentration of MazF-SEA for each probe was determined from preliminary experiments (Table S5). Then, MazF-SEA and several concentrations of probes were incubated at 37 ºC in 20 μl of MazF reaction buffer. UACU or UACC probes (0.20, 0.35, 0.50, 0.75, 1.0, 1.5, and 2.0 μM) and UACG or UACA probes (0.10, 0.15, 0.20, 0.35, 0.50, 0.75, 1.0, and 1.5 μM) were used. Fluorescence intensity was recorded every 30 s using a Light Cycler 480 (Roche). To convert the fluorescence intensity into the concentration of cleaved oligonucleotides, the normalized fluorescence intensity of MazF-cleaved oligonucleotides was multiplied by the initial concentration of the oligonucleotides. Subsequently, temporal changes in the concentration were fitted using Kaleida Graph 4.5.0 (Synergy Software).

### Data analysis

Plots and graphs were generated using R v4.2.1 (47) with packages tidyverse v1.3.1 (48) and ggplots v3.3.6 (49).

## Data availability

The GenBank accession numbers are as follows: MazF-SEA (WP_000373725.1), MazE-SEA (WP_000288813.1), MazF-dra (WP_010887062.1), 1500-1 (AB610949.1), L1500-1 (LC659330), H1500-1 (LC659334), 2000-1 (AB610950), L2000-1 (LC659331), and H2000-1 (LC659335). The sequence data were deposited to the DDBJ database under accession numbers DRA015035 (MazF-SEA WT) and DRA015036 (MazF-SEA R73L mutant).

## Supporting information

This article contains supporting information.

## Conflict of interest

The authors declare no conflicts of interest regarding the contents of this article.
