# Supplementary material for: Arg-73 of the RNA endonuclease MazF in Salmonella enterica subsp. arizonae contributes to guanine and uracil recognition in the cleavage sequence
Source: J Biol Chem. 2024 Jan 9;300(2):105636. doi: 10.1016/j.jbc.2024.105636 (PMC10864209; doi:10.1016/j.jbc.2024.105636)
Supplement: Supplemental Figure [file mmc1.pdf]

Supplementary Materials.

Arg-73 of the RNA endonuclease MazF in *Salmonella enterica* subsp. *arizonae* contributes to guanine and uracil recognition in the cleavage sequence

Takuma Okabe[1,2], Rie Aoi[1,2], Akiko Yokota[2], Hiroko Tamiya-Ishitsuka[2], Yunong Jiang[2,3], Akira Sasaki[2], Satoshi Tsuneda[1*], and Naohiro Noda[1,2,4*]

[1] Department of Life Science and Medical Bioscience, Waseda University, Tokyo, Japan
[2] Biomedical Research Institute, National Institute of Advanced Industrial Science and Technology (AIST), Ibaraki, Japan
[3] Graduate School of Comprehensive Human Sciences, University of Tsukuba, Ibaraki, Japan
[4] School of Integrative and Global Majors, University of Tsukuba, Ibaraki, Japan

[*]**Corresponding author:**
Satoshi Tsuneda, E-mail: stsuneda@waseda.jp
Naohiro Noda, E–mail: noda-naohiro@aist.go.jp

Running Title: Arg-73 of MazF-SEA crucial for specific cleavage activity

Keywords: endoribonuclease, enzyme kinetics, fluorescence resonance energy transfer (FRET), protein engineering, RNA‐protein interaction, *Salmonella enterica*, structural model, substrate specificity.

Funding: This work was supported by Japan Society for the Promotion of Science (JSPS) KAKENHI Grant Number JP19K06555 and the Tokyo Kasei Chemical Promotion foundation.

Conflicts of interest: The authors declare no conflicts of interest regarding the contents of this article.

Supplementary Information

Figure S1. Fluorometric assay of WT MazF-SEA with MazE-SEA.
Figure S2. Comparison between MazF-SEA and MazF-bs residues interacting with RNA.
Figure S3. Purification of MazFs.
Figure S4. Fluorometric assay of MazF-SEA mutants.

Table S2. Kinetic parameters of WT and R73L mutant MazF-SEA.
Table S4. Sequences of barcode RNA and RNA/DNA chimeric oligonucleotides.
Table S5. Concentration of enzymes used in the evaluation of kinetic parameters for each substrate.

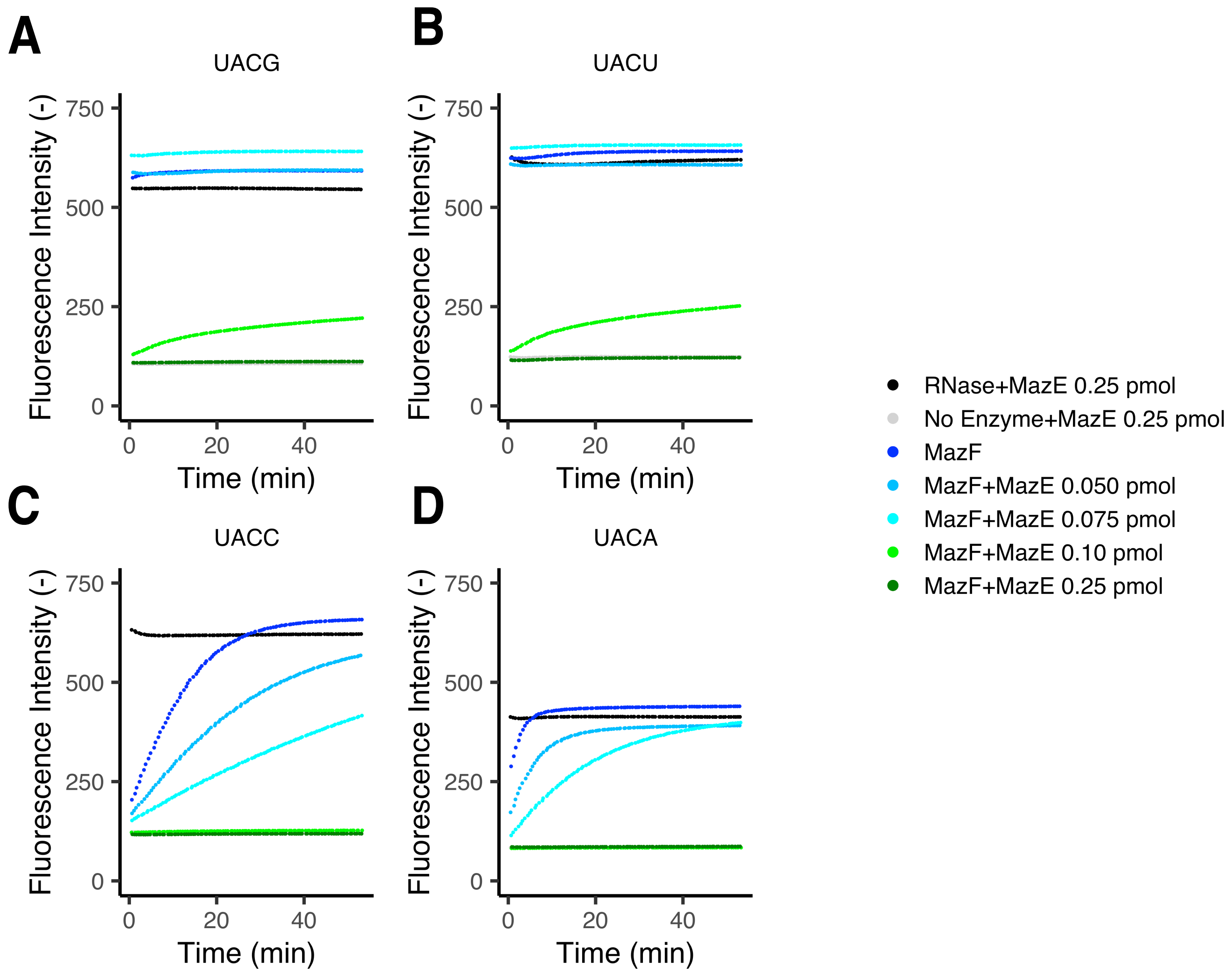

Figure S1. Fluorometric assay of WT MazF-SEA with MazE-SEA.
MazE-SEA (0, 0.050, 0.075, 0.10 or 0.25 pmol) was reacted with 100 ng of RNase A, no enzyme, or 0.5 pmol of WT MazF-SEA for 10 min at room temperature. Then, these reacted mixtures were reacted with 20 pmol of the RNA/DNA chimeric oligonucleotide probes containing either (A) UACG, (B) UACU, (C) UACC, or (D) UACA at 37 ºC. Fluorescence intensity was recorded every 30 s using a Light Cycler 480. RNase A cleaved all probes regardless of MazE, while MazE-only samples did not cleave any probes. MazE-SEA inhibited the cleavage activities of WT in a dose-dependent manner for all probes. This result would have confirmed that the sequence-specific cleavage of RNA was due to recombinant proteins and not to contaminants. The grey labeled data points (No enzyme+MazE) are behind the dark green data points in all graphs.

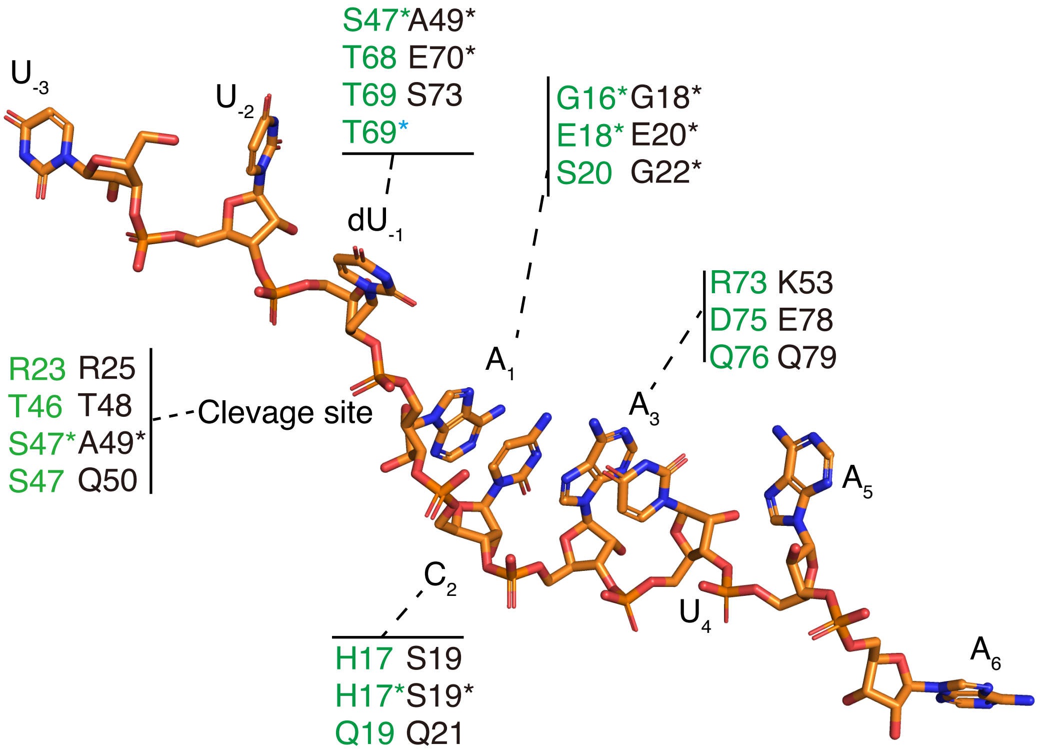

Figure S2 Comparison between MazF-SEA and MazF-bs residues interacting with RNA.
The RNA ($U_{-3}U_{-2}dU_{-1}A_1C_2A_3U_4A_5A_6$) structure is extracted from the co-crystal structure of RNA-bound MazF-bs (PDB:4mdx). The residues of MazF-SEA (green) interacting with four bases of its cleavage sequences (U_{-1}^$A_1C_2G_3/U_3$) and the cleavage site were predicted by aligning with the co-crystal structure of RNA-bound MazF-bs. Moreover, MazF-SEA residues were compared with MazF-bs residues (black) which form hydrogen bonds with each base of its cleavage sequence (U_{-1}^$A_1C_2A_3U_4$). Asterisks represent residues where the main chain interacts with the RNA.

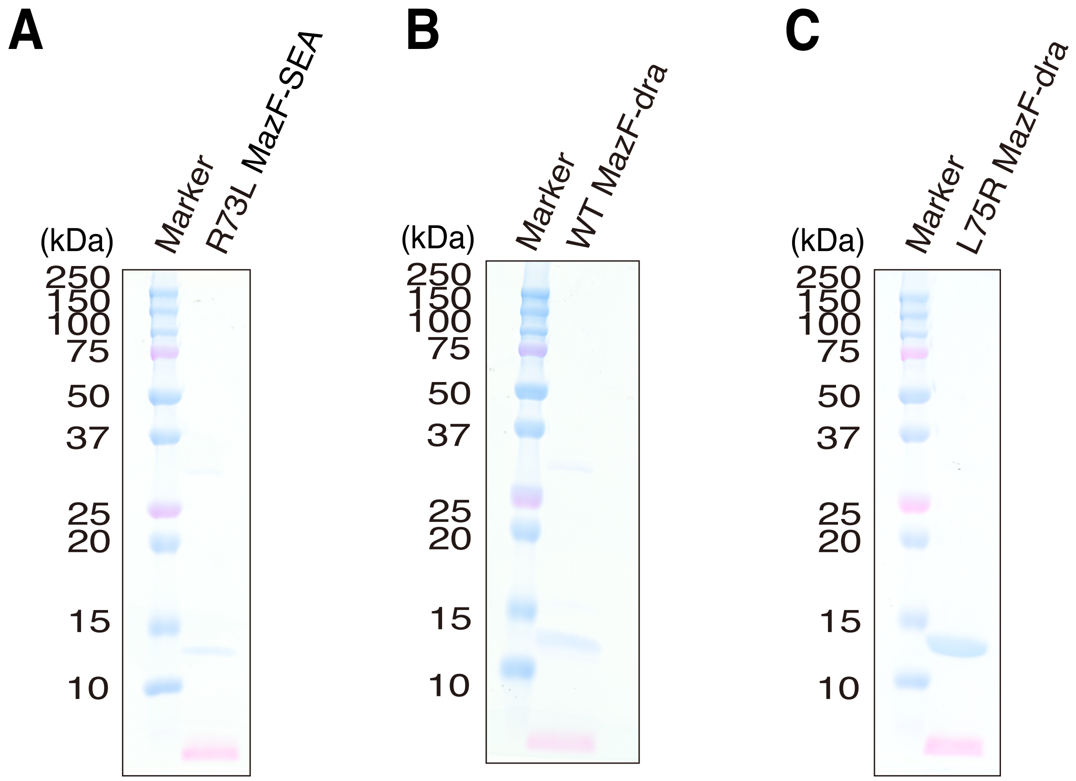

Figure S3. Purification of MazFs.
(A) R73L mutant MazF-SEA, (B) WT MazF-dra, and (C) L75R mutant MazF-dra were evaluated using SDS-PAGE. The theoretical molecular weights of R73L mutant MazF-SEA, WT MazF-dra, and L75R mutant MazF-dra are 12.8, 13.5, and 13.5 kDa, respectively.

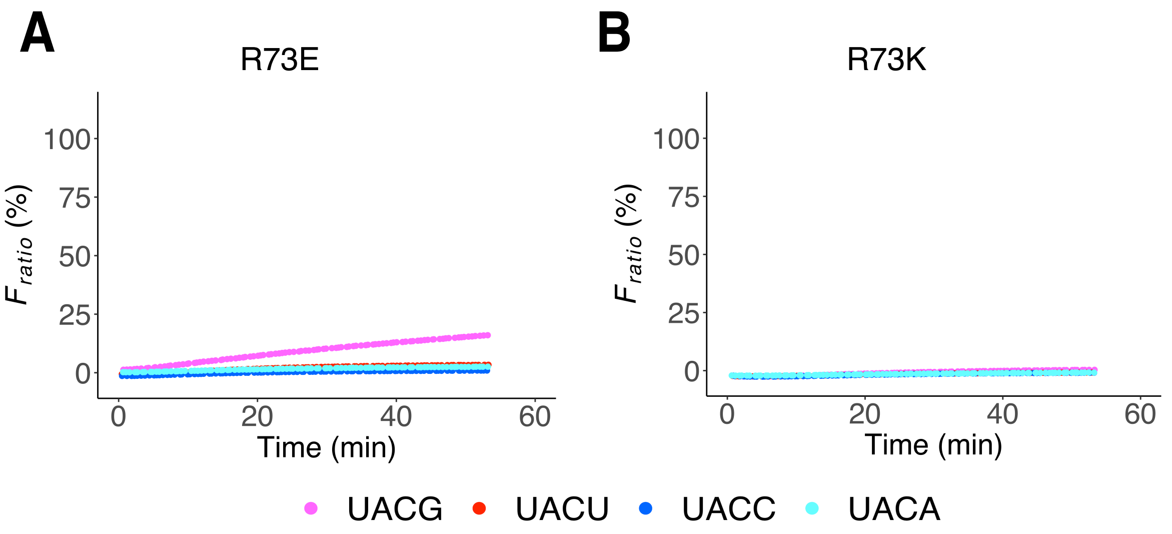

Figure S4 Fluorometric assay of MazF-SEA mutants. RNA/DNA chimeric oligonucleotides containing UACG (pink), UACU (red), UACC (blue), or UACA (cyan) were incubated with (A) 0.5 pmol of R73E mutant MazF-SEA or (B) 0.5 pmol of R73K mutant MazF-SEA at 37 ºC. Fluorescence intensity was recorded every 30 s using Light Cycler 480. The excitation and detection wavelengths were 465 nm and 510 nm, respectively.

Table S2. Kinetic parameters of WT and R73L mutant MazF-SEA.

Probes	WT			R73L		
	K_M (μM)	k_{cat} (s^{-1})	k_{cat}/K_M ($\mu M^{-1}s^{-1}$)	K_M (μM)	k_{cat} (s^{-1})	k_{cat}/K_M ($\mu M^{-1}s^{-1}$)
UACG	0.17 ± 0.02	2.6 ± 0.3	16 ± 3	0.26 ± 0.05	0.095 ± 0.013	0.38 ± 0.08
UACU	0.47 ± 0.17	2.1 ± 0.6	4.8 ± 1.7	2.1 ± 0.8	0.54 ± 0.13	0.27 ± 0.05
UACC	0.23 ± 0.07	0.026 ± 0.008	0.12 ± 0.07	3.2 ± 1.6	0.22 ± 0.08	0.072 ± 0.009
UACA	0.32 ± 0.11	0.060 ± 0.012	0.21 ± 0.12	0.87 ± 0.48	0.10 ± 0.03	0.12 ± 0.03

Table S4. Sequences of barcode RNA and RNA/DNA chimeric oligonucleotides.

Name	Sequence[1]
Barcode RNA	GCUGAUGGCGAUGAAUGAACACU GCGUUUGCUGGCUUUGAUGAAA
UACG	aaaaaUACGaaaaa
UACU	aaaaaUACUaaaaa
UACC	aaaaaUACCaaaaa
UACA	aaaaaUACAaaaaa

[1] Uppercase letters represent RNA sequence. Lowercase letters indicate DNA sequence.

Table S5. Concentration of enzymes used in the evaluation of kinetic parameters for each substrate.

	WT (nM)	R73L (nM)
UACG	0.25	7.5
UACU	0.25	7.5
UACC	12.5	12.5
UACA	7.5	12.5
